# Prevalence and treatment of retrograde peri-implantitis: a retrospective cohort study covering a 20-year period

**DOI:** 10.1007/s00784-020-03769-5

**Published:** 2021-01-14

**Authors:** Bianca Di Murro, Luigi Canullo, Giorgio Pompa, Carlo Di Murro, Piero Papi

**Affiliations:** 1 Oral Surgery Unit, Department of Oral and Maxillo-Facial Sciences, ‘Sapienza’ University of Rome, Via Caserta 6, 00161 Rome, Italy; 2 Private Practice, Rome, Italy

**Keywords:** Peri-implantitis, Dental implants, Tooth extraction, Periapical diseases, Periapical granuloma

## Abstract

**Objectives:**

The aims of this retrospective study were to report data on the prevalence of retrograde peri-implantitis (RPI) in a single-center in a 20-year observation period and to evaluate implant survival after surgical treatment.

**Materials and methods:**

A retrospective cohort study was conducted screening all patients who underwent implant treatment in a private practice. Patients were enrolled if they had one or more implants showing a radiolucency around the implant apex, without implant mobility. Furthermore, clinical symptoms of RPI and days from symptoms’ appearance after implant placement were also collected, as well as periodontal and endodontic status of nearby teeth. All patients were treated with the same surgical approach: antibiotic therapy, mechanical curettage, chemical decontamination and xenograft application.

**Results:**

Out of the 1749 implants placed, only 6 implants were classified as affected by RPI, with a prevalence of 0.34%. Clinical symptoms of RPI (pain, swelling, dull percussion or fistula presence) varied among patients and were reported after a mean period of 51.83 ± 52.43 days.

**Conclusions:**

RPI was successfully treated with surgical curettage and bone substitute application and all implants are still in place after a mean follow-up of 8.83 ± 5.34 years.

**Clinical relevance:**

Bacteria from teeth with failed endodontic treatment or residual lesions might be reactivated by drilling for implant osteotomy, with subsequent colonization of the implant apex and possible failure before prosthetic loading. Therefore, it might be recommended to take a periapical x-ray at implant placement and after 6–8 weeks in order to intercept RPI before prostheses delivery.

## Introduction

Implant-supported prosthetic rehabilitation is considered a predictable and successful option for replacing missing teeth in partially and fully edentulous patients [1–3]. However, the widespread diffusion of dental implants has been associated, in the last 30 years, with the rise of mechanical [4–7] and biological complications [8, 9], divided in peri-implant mucositis and peri-implantitis [10]. Peri-implantitis is an irreversible plaque-related inflammatory lesion and the first cause of late implant failure [11]. It is defined, by the 2017 World Workshop on the Classification of Periodontal and Peri-Implant Diseases and Conditions, as a pathological inflammation of peri-implant tissues with progressive loss of supporting bone detected radiographically [12]. The prevalence of peri-implantitis is still controversial, depending primarily on the case definition adopted: a recent systematic review found out that around 23% of dental implants are affected by peri-implantitis and 43% by mucositis [13]. At the same time, due to the heterogeneity of the case definition, a recent systematic review [14] downgraded the peri-implantitis rate 18.5% at the patient level and 12.8% at the implant level.

Another disease, called retrograde/periapical peri-implantitis (RPI) [15], was firstly described in 1992. The RPI affects only the peri-apical portion of the implant and is detected radiographically as a radiolucent area, without pathological probing and signs of marginal bone loss [16]. A very low prevalence (0.26–1.86%) has been reported for RPI [17]; therefore, considering also the few articles available in the scientific literature on the topic, the pathology is still relatively unknown among clinicians [18]. Zhou et al. reported a prevalence of RPI of 7.8% in cases of dental implants placed adjacent to teeth with endodontic periapical lesions [19], while Lefever et al. described odds ratio (OR) of developing RPI ranging from 7.2 to 8 in cases of endodontic pathology on the extracted or neighbouring tooth [20].

There is no consensus about RPI aetiology: the main possible causes hypothesised are implant insertion in a site with a pre-existing unhealed infection or inflammation [21], implant placement in a site that previously housed an endodontic treated tooth with further bacteria reactivation [22, 23], pulpal/periapical endodontic lesions at adjacent teeth or bone overheating during implant drilling [24, 25]. Furthermore, also, implant placement in a longer prepared osteotomy site has been reported as a probable cause of asymptomatic periapical lesions at the implant site [16, 21]. The RPI is characterised by progressive bone loss at the apical part of the implant, detected radiographically, in the first weeks up to 4 years after implant placement [15], without radiological alterations of peri-implant marginal bone levels and pathological probing pocket depths [26].

Clinical findings are not always present and can include pain, dull percussion, swelling, tenderness, redness and a fistulous sinus tract site at the buccal apical part of the implant [17].

Regarding the treatment of RPI, there is no clear consensus in literature [20, 27]: therapeutic modalities based only on antibiotic therapy with/without endodontic treatment of the adjacent tooth [28] have been described, together with surgical approaches with the aim to eliminate the inflammatory process and allow the re-osseointegration of the apical part of the implant [29]. The surgical treatment usually includes surgical/chemical debridement of the apical implant site with/without bone regeneration procedures and with/without the resection of the implant apex [29–31].

The aims of this retrospective study were to report data on the prevalence of retrograde peri-implantitis in a single-center during a 20-year observational period (1999–2019) and to evaluate implant survival following a standardized surgical procedure.

## Material and methods

### Study design

A retrospective cohort study was conducted screening all medical and radiographic records of patients who underwent implant treatment in a private practice setting located in Rome, Italy, between January 1, 1999, and December 31, 2019. Due to its retrospective nature, the study was approved by the Institution Review Board (IRB) of the Department of Oral and Maxillo-Facial Sciences, at ‘Sapienza’ University of Rome (Ref. 21/2020). All patients agreed to be included in the study, signed the informed consent form and agreed to x-ray publication, according to the latest version of the World Medical Declaration of Helsinki (2013). The study was reported in accordance with the Strengthening the Reporting of Observational studies in Epidemiology (STROBE) guidelines for cohort studies.

### Inclusion and exclusion criteria

Patients were enrolled in this retrospective study if they had one or more implants showing a radiolucency around the implant apex, without implant mobility.

### Data recording

For each patient with a radiographic diagnosis of RPI, the following variables were collected: sex, age, smoking habits, periodontal status (presence or absence of periodontitis) and reason for tooth extraction prior to implant placement. Furthermore, clinical symptoms of RPI (pain, swelling, dull percussion or fistula presence) and days from symptoms’ appearance after implant placement were also collected, as well as periodontal and endodontic status of nearby teeth.

Periapical x-rays taken with the long-cone parallel technique and a standardized film holder (Rinn Centratore XCP, Dentsply, Rome, Italy) prior to implant placement, at RPI diagnosis, 3 months after surgical treatment and at the latest follow-up available were collected for each patient included in the study.

### Surgical procedure

After diagnosis of RPI through radiographic examination and symptoms presentation, a sensibility test was performed at the adjacent teeth of implants involved to assess their vitality and, therefore, the possible endodontic origin of RPI.

An antibiotic treatment was prescribed to all patients: a combination of amoxicillin 500 mg (Zimox®, Pfizer, New York, USA) and metronidazole 250 mg (Flagyl®, Pfizer, New York, USA) 3 times/day for 1 week, starting 1 day before surgery. The same surgical approach was performed in all cases included by the same operator (CDM).

At the beginning of the procedure, patients were instructed to rinse for 1 min with chlorhexidine gluconate 0.12%. Surgery was performed under local anaesthesia, with a sterile operating field. A mucoperiosteal flap was raised to gain access at the affected area by using a 15c scalpel blade (Hu-Friedy, Chicago, IL, USA), performing intrasulcular incisions at the implant and the distal tooth and a releasing incision at the mesial tooth. Meticulous mechanical debridement and degranulation of the bone in the periapical implant site were performed using Lucas spoon and Gracey curettes. Ultrasonic devices and carborundum burs were used to polish the spiral convexity of the implant surface. A chemical detoxification was performed, by applying chlorhexidine gluconate 0.2% for 2 min on the titanium surface, and then, by rinsing abundantly the site with sterile saline solution 0.9%. Prior to flap closure with non-absorbable sutures, the cavity was filled by using small particles of deproteinized bovine bone material (Bio-oss, Geistlich AG, Wolhusen, Switzerland). Due to the self-containment nature of all defects, a membrane was not applied over the bone substitute in all patients. The adjacent teeth remained untouched during the procedure. Standard postoperative instructions were prescribed together with rinsing with 0.2% chlorhexidine digluconate 2 times a day and to avoid tooth brushing in the area for 14 days, with ibuprofen 600 mg (Brufen, Abbott, Verona, Italy) prescribed to be taken as needed. Sutures removal was performed after 14 days.

### Follow-up

Prosthetic treatment including the application of a temporary crown and the final rehabilitation was performed by the same dentist (CDM) according to the specific treatment plan. Patients were enrolled in a maintenance program and recalled every 3 months for oral hygiene procedures. An implant in place at the respective follow-up visit was considered surviving implant.

### Peri-implant parameters

For each implant affected by RPI, the following clinical measurements were also recorded at six sites per implant by using a periodontal probe (PCP-Unc 15, Hu-Friedy®, Chicago, Illinois, USA) with a light force (approximately 0.15 N), without anaesthesia by the same trained calibrated operator (CDM) at day of prostheses delivery (baseline), after 3 years and at the latest follow-up available:

- Probing pocket depth (PPD), measured in millimetres, is the distance from the mucosal margin to the bottom of the probable pocket
- Plaque index (PI) recorded with dichotomic values (present/absent)
- Bleeding on probing (BOP) recorded with dichotomic values (present/absent)

Furthermore, full mouth plaque score (FMPS) and full mouth bleeding score (FMBS) were recorded at baseline, after 3 years and at the latest follow-up available.

### Statistical analysis

Data were evaluated using a standard statistical analysis software (version 20.0, Statistical Package for the Social Sciences, IBM Corporation, Armonk, NY, USA). A database was created using Excel (Microsoft, Redmond, WA, USA). Descriptive statistics (mean, standard deviations and range) were computed for each continuous variable collected, while frequency was reported for categorical variables.

## Results

### Screening process

During the 20-year observational period (1999–2019), a total of 1749 dental implants were placed in 708 patients, with a mean of 2.47 implants per patient: they were all tissue-level implants with a sandblasted acid-etched surface (SLA) (Institut Straumann AG, Basel, Switzerland), and a transmucosal healing protocol was adopted in all cases. Dental implants were placed adopting a delayed approach several months after teeth extractions, following proper manufacturers’ instructions and checking intraoperatively correct buccal-oral position of the implant and integrity of the facial bone wall.

An accurate anamnesis was recorded for all patients, collecting pre-, intra- and post-operative clinical and radiographic data. Patients’ data collected included sex, age and reasons for tooth loss. For each implant, length, diameter, years of functional loading, implant location (maxilla or mandible) and type of prostheses (single crown or multiple unit) were recorded (Table 1).

**Table 1:** Demographic characteristics of dental implants placed in the study period (1999–2019)

Variable	Implants (*n* = 1749)	Early Implant failure	Implants affected by RPI
*Reason for tooth loss*			
Caries	589	31	0
Periodontitis	618	34	2
Fracture	230	9	1
Endodontic failure	274	22	3
Agenesis	38	2	0
*Implant site*			
Maxilla	925	42	2
Mandible	824	56	4
*Implant length*			
8 mm	307	25	0
10 mm	498	18	4
12 mm	601	20	2
14 mm	343	35	0
*Implant diameter*			
Wide neck	554	59	2
Regular neck	1195	39	4
*Type of prosthesis*			
Single crown	902	52	4
Multiple unit	847	46	2

*RPI* retrograde peri-implantitis

Out of all implants inserted in the study period, 98 failed during the osseointegration phase (5.6%), while only 6 of the remaining 1651 implants showed a radiolucency surrounding the implant apex and were classified as affected by RPI, with a prevalence of 0.34% (Table 1).

### RPI sample

All patients were male, with a mean age of 54.66 ± 9.41 years (range: 38–65 years) and implants affected constituted 31.6% of the total implants placed in these patients (*n* = 6/19), with every subject having all the other dental implants classified as clinically healthy.

**Fig. 1:**
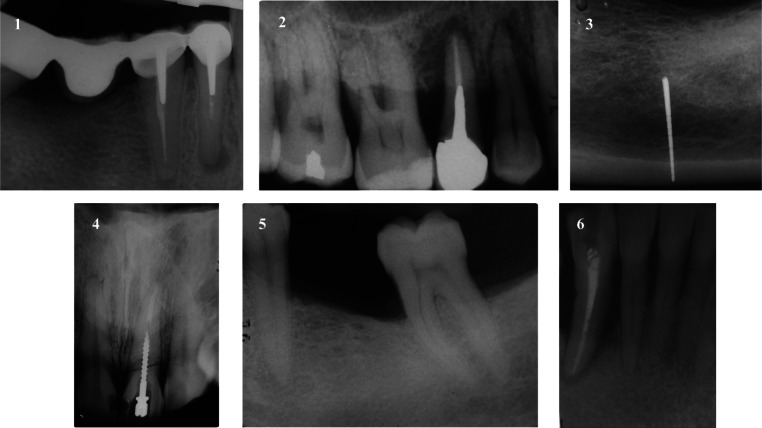
Periapical x-rays of the 6 patients prior to implant placement. In the first patient, teeth were extracted for endodontic treatment failure, they both presented periapical lesions. In the second patient, tooth 1.5 was extracted for endodontic failure. The third patient had his tooth (3.7) extracted for severe periodontitis. In the fourth patient, tooth 2.1 had a horizontal fracture and a crack over the root due to the endocanalar post displacement. Fifth patient had his tooth (3.6) extracted for periodontitis. The sixth patient lost his tooth (4.3) due to a severe endodontic-periodontal lesion; the tooth was necrotic and presented a visible periapical lesion

**Fig. 2:**
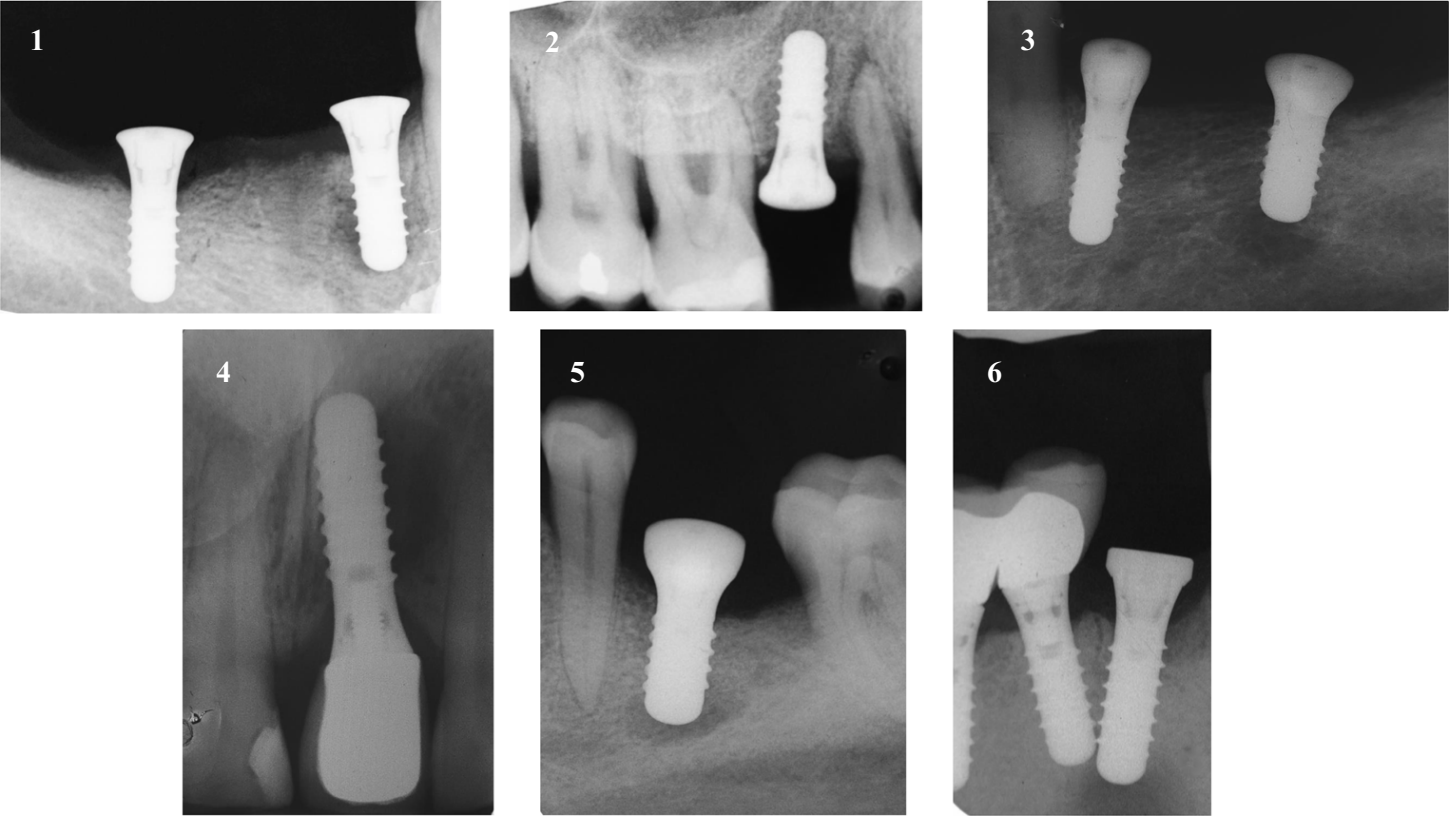
The RPI lesions in the six implants. The first patient described an acute pain in the apical zone of the mesial implant 5 days after implant placement. The second patient reported the same symptoms after 51 days. The third patient presented pain while blowing nose and sneezing in the area of the distal implant after 25 days. The fourth patient came to our observation describing to feel a soft and painful point on the palatal aspect of the implant after 165 days. The fifth patient presented pain, swelling and a fistulous tract over the vestibular aspect of the implant periapical bone after 29 days. The sixth patient showed swelling and redness over the mesial implant area and described pain on percussion 36 days after implant placement

**Fig. 3:**
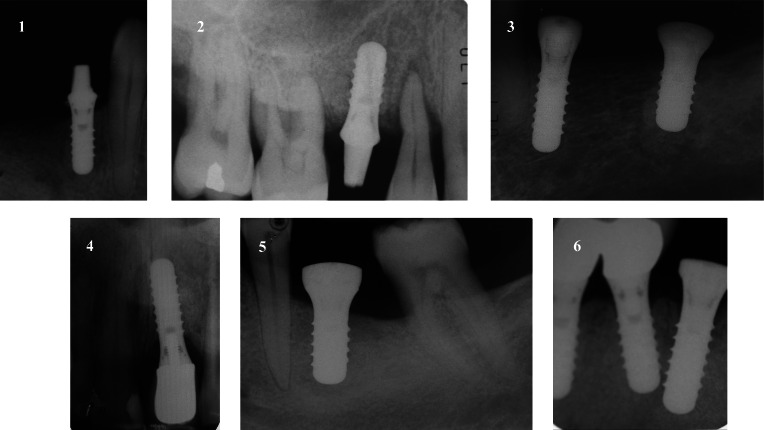
The six implants at a radiographic examination three months after surgical treatment of RPI

Reasons for extraction prior to implant placement were endodontic treatment failure (2 teeth), severe periodontitis (2 teeth), the endodontic-periodontal lesion (1 tooth) and root fracture (1 tooth) (Fig.1). All implants were placed 4 months after tooth extraction, except for two cases, where teeth were extracted more than 3 years prior to implant placement. No implant was placed adjacent to endodontically treated teeth, while four patients were classified as periodontitis cases based on the latest 2017 World Workshop classification [32]. In three cases, implants affected had both adjacent teeth, in two cases mesially a tooth and distally an implant and the remaining case was a distal element of an implant-supported fixed prosthodontics (Fig. 1). Patients reported symptoms after a mean period of 51.83 ± 52.43 days (range: 5–165 days) from implant placement: RPI was diagnosed after prostheses delivery just in one case (Fig. 2). Table 2 describes the detailed characteristics of implants diagnosed with RPI. Following RPI surgical treatment, the inflammatory process was eliminated and no adverse reactions or complications were recorded. Three months after surgery (Fig. 3), the prosthetic treatment was completed, and all implants were successfully restored: three single crowns and three multiple units.

**Table 2:** Detailed characteristics of dental implants with retrograde peri-implantitis

Variable	Case 1	Case 2	Case 3	Case 4	Case 5	Case 6
Sex	M	M	M	M	M	M
Age	50	52	65	38	65	58
Smoking habits	No	Yes	No	No	No	Yes
Periodontitis	No	Yes (stage 3, grade B)	Yes (stage 3, grade A)	No	Yes (stage 3, grade A)	Yes (stage 3, grade B)
Implant position	4.4	1.5	3.7	2.1	3.6	4.3
Reason for tooth loss	Endodontic failure	Endodontic failure	Periodontitis	Fracture	Periodontitis	Endo-periodontal lesion
Implant length	10	10	10	12	10	12
Implant diameter	4.1	4.1	4.8	4.1	4.8	4.1
Symptoms		51				
Days until appearance following implant insertion	5		25	165	29	36
Constant pain	Yes	No	No	No	Yes	No
Dull percussion	No	Yes	Yes	No	Yes	Yes
Swelling	No	No	No	Yes	Yes	Yes
Fistulous tract	No	No	No	No	Yes	No
Mesial neighbour	Vital	Vital	None	Vital	Vital	Vital
Distal neighbour	None	Vital	None	Vital	Vital	Implant
Total implants (n)	5	4	2	2	2	4
Follow-up (years)	9	8	6	20	7	3

**Fig. 4:**
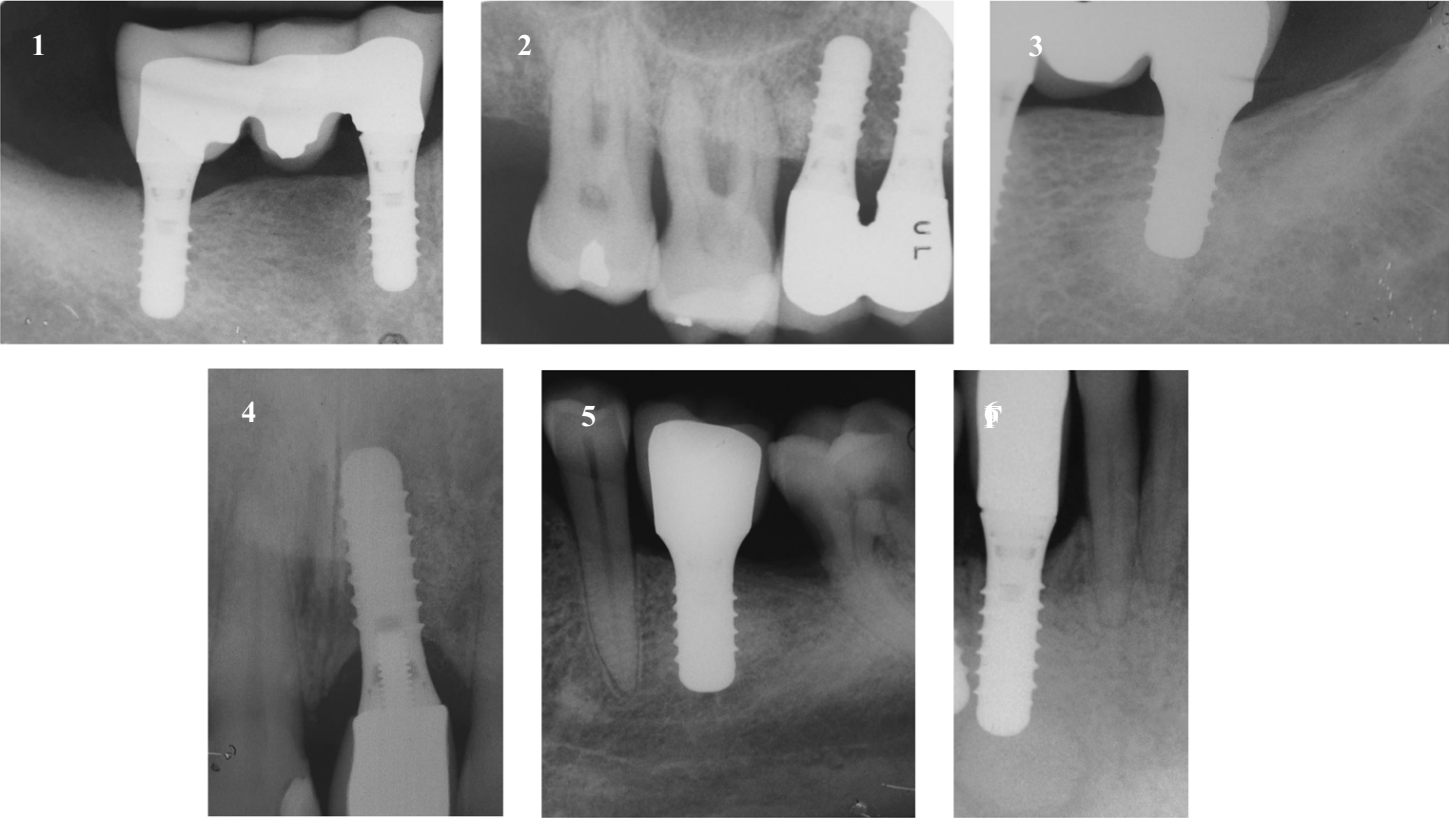
The latest follow-up available after RPI treatment. Patient 1: 9 years. Patient 2: 8 years. Patient 3: 6 years. Patient 4: 20 years. Patient 5: 7 years. Patient 6: 3 years. Neighbouring teeth remained untreated and vital throughout the observation period, except for one patient (2) who lost one adjacent tooth due to severe periodontitis several years after surgical treatment and was, then, treated with an implant

No implant was lost after treatment: a 100% survival rate was detected after a mean follow-up of 8.83 ± 5.34 years (range: 3–20 years) (Fig. 4). Healing was uneventful in all cases treated, no adverse reactions were reported and peri-implant clinical parameters collected throughout the follow-up period are reported in Table 3. Adjacent teeth remained untreated, except for one patient who lost one adjacent tooth due to severe periodontitis several years after surgical treatment and was, then, treated with an implant (Fig. 4).

**Table 3:** Clinical parameters of dental implants with retrograde peri-implantitis

Variable	Case 1	Case 2	Case 3	Case 4	Case 5	Case 6
Probing pocket depths (PPD) implant site						
PPD-baseline	3.33 ± 0.51	3.8 ± 1.16	3.8 ± 0.75	3.33 ± 0.51	3.33 ± 0.51	4 ± 0
PPD, 3 years	4 ± 1.09	3.16 ± 0.98	4 ± 1.09	3.16 ± 0.75	3.8 ± 0.75	4.3 ± 1.03
PPD, the latest follow-up	3.8 ± 0.75	3.33 ± 0.51	4 ± 0	4.16 ± 0.98	3.8 ± 1.16	4.3 ± 1.36
Bleeding on probing (BOP)						
BOP, baseline	–	–	–	–	–	–
BOP, 3 years	–	–	+	–	+	–
BOP, the latest follow-up	–	–	–	–	–	–
Plaque index (PI) implant site						
PI, baseline	–	–	–	–	–	+
PI, 3 years	–	–	+	–	–	–
PI, the latest follow-up	–	+	–	–	–	–
Full mouth bleeding score (FMBS)						
FMBS, baseline	20%	24%	23%	16%	22%	25%
FMBS, 3 years	22%	23%	25%	14%	22%	25%
FMBS, the latest follow-up	20%	20%	23%	12%	20%	25%
Full mouth plaque score (FMPS)						
FMPS, baseline	16%	20%	23%	16%	20%	25%
FMPS, 3 years	20%	20%	23%	10%	18%	23%
FMPS, the latest follow-up	20%	20%	20%	18%	16%	23%

## Discussion

The aims of this retrospective study were to report data on the prevalence of RPI in a single-center during a 20-year observational period (1999–2019) and to evaluate implant survival after surgical approach. Among the 1749 dental implants placed in the study period, only six were affected by RPI, with a prevalence of 0.34%. All the implants were surgically treated with curettage and xenograft application and they are still in place, with a survival rate of 100% after a mean follow-up of 8.83 ± 5.34 years. Speculating on the possible aetiology of RPI in implants included in the present study, a common finding was that there were no implants with adjacent endodontically treated teeth. According to the classification proposed by Sussman and Moss in 1993, RPI is an endodontic implant pathology, divided in type 1 (implant to tooth lesion), which occurs when the implant placement results in the devitalization of an adjacent tooth, and type 2 (tooth to implant lesion), when an apical lesion from a neighbouring endodontically treated tooth contaminates the implant [25].

The original classification was implemented by Sarmast et al. in 2016, with the inclusion of two additional classes: type 3 and type 4 [29]. Type 3 is related to apical implant lesion developing in case of improper implant angulation (i.e., outside the bone cortex). Type 4 is related to apical implant lesion developing for residual microorganisms (viruses or bacteria) or bone infection reactivation, with a non-osseointegration of the apical implant zone and its contamination.

The 6 cases reported in the present study might all be ascribed to class 4 of the Sarmast classification [29]. In four cases, implants were placed in sites with previously endodontically treated teeth, with two teeth affected also by apical periodontitis and one with a periapical lesion. In the remaining two cases, patient teeth were extracted for severe periodontitis more than 3 years prior to implant placement.

A recent systematic review [23] evaluated the histopathological and microbiological findings associated with RPI. In the six studies included, 21/30 dental implants with a RPI diagnosis were associated with failed endodontic treatment, apical periodontitis or remaining infected roots at the implant site. In these cases, microbiological analysis of samples collected revealed the following bacteria: Porphyromonas gingivalis, Corynebacterium, Streptococcus and *Klebsiella pneumoniae*. Siqueira et al. [33] reported that *Enterococcus faecalis* is the most common bacteria found at the apex of endodontically treated teeth; furthermore, also, Prevotella intermedia, Fusobacterium Nucleatum and Porphyromonas gingivalis have been discovered after endodontic therapy [34]. These bacteria might remain encapsulated in cancellous bone after the extraction of teeth with failed endodontic treatment and might be reactivated by drilling for implant osteotomy, with subsequent colonization of the implant apex [35]. A retrospective study [20] reported an odds ratio (OR) of 7.2 for a tooth with an endodontic history to develop RPI, even in absence of periapical lesions. Observations from the present study are in accordance with previous findings: an association between endodontic therapy and RPI was present in four cases included in the present study. However, RPI was diagnosed also in two cases of dental implants placed in sites without a previous endodontic treatment or endodontically treated adjacent teeth. Another possible explanation for implant apex contamination could be the presence of residual lesions (granulomas, residual apical cysts, root remnants) of the extracted teeth [20, 23] or bone overheating during implant drilling [25]. Even in cases of appropriate curettage of the alveolar cavity, bacteria could remain encapsulated and be reactivated by implant drilling [35]. In the present study group, patients had teeth extracted from 4 months to several years prior to implant placement and pre-operative periapical radiographs did not show any radiolucent lesion. All patients came to observation with symptoms; therefore, periapical x-rays were taken, and lesions diagnosed. Asymptomatic RPI lesions diagnosed by routine radiographic examinations have been also reported: this might be an explanation on the relatively low prevalence and knowledge of RPI compared to marginal peri-implantitis [30]. Hence, RPI generally occurs in the first weeks after placement and asymptomatic untreated lesions could lead to implant mobility and lack of osseointegration, with early implant failure before prosthetic loading and an underestimation of the disease. Therefore, it might be recommended to take a periapical x-ray at implant placement and after 6–8 weeks before prostheses delivery in order to intercept and to early detect signs of RPI [18]: a radiolucent lesion surrounding the implant apex should always alert the clinician, with the exception of implant overdrilling.

All patients were treated with the same surgical approach: antibiotic therapy, curettage and removal of the lesions, chemical decontamination and bone regeneration. To the best of the authors’ knowledge, only four studies [20, 26, 31, 36] reported data on the implant survival rate and follow-up after treatment: cumulative survival rate (CSR) ranged from 67.5 to 97.4%, with a mean follow-up from 72 months to 4.54 years. In our study, we reported for the first time a CSR of 100%, with the maximum follow-up available in literature, with a mean observation period of 8.83 ± 5.34 years. Even if the management of RPI is still unclear, removal of all granulation tissue, with a careful decontamination of the implant apex and following bone graft, seems to arrest bone loss progression.

Main limitations of this study are represented by its retrospective nature and the limited sample enrolled; however, studies on RPI are usually case reports with a smaller follow-up and a high drop-out rate. In the present study group, there were no drop-outs or loss to follow-up and the same clinician performed implant placement, RPI diagnosis and surgical management. Another limitation is the impossibility to exclude that no other implant suffered from RPI during the study period, since not all patients followed the strict monitoring process after implant placement and some early failures could be, in reality, caused by missed retrograde peri-implantitis.

## Conclusions

Prevalence of retrograde peri-implantitis is extremely low: among the 1749 dental implants placed in the study period, only six were affected by RPI (0.34%). All the implants were surgically treated with curettage and xenograft application and they are still in place, with a survival rate of 100% after a mean follow-up of 8.83 ± 5.34 years (range: 3–20 years). Based on the analysis of patients’ characteristics, it can be speculated that bacteria from teeth with failed endodontic treatment or residual lesions were reactivated by drilling for implant osteotomy, with subsequent colonization of the implant apex. Even if, in absence of histological samples, the authors cannot exclude a mechanical aetiology caused by bone overheating during implant drilling. Therefore, within the limitations of the study, it could be cautiously concluded that RPI can be predictably and successfully treated with surgical curettage and bone substitute application. Further studies, with a longitudinal design and larger sample, are needed to confirm these clinical findings.
